# Immobilized aptamer on gold electrode senses trace amount of aflatoxin M1

**DOI:** 10.1007/s13204-017-0629-0

**Published:** 2017-11-13

**Authors:** Amit Kumar Pandey, Yudhishthir Singh Rajput, Rajan Sharma, Dheer Singh

**Affiliations:** 1 Animal Biochemistry Division, National Dairy Research Institute (NDRI), Karnal, Haryana 132001 India; 2 Dairy Chemistry Division, National Dairy Research Institute (NDRI), Karnal, Haryana 132001 India

**Keywords:** Electrochemical aptasensor, Aflatoxin M1, Aptamer, Square wave voltammetry

## Abstract

An electrochemical aptasensor for detection of trace amounts of aflatoxin M1 was developed. This required immobilization of aptamer on screen printed gold electrode comprising of working electrode, counter electrode and reference electrode and was achieved by sequentially layering dithiodipropionic acid, streptavidin and biotinylated-tetraethylene glycol-aptamer. Immobilization of aptamer was monitored by cyclic voltammetry. Peak current in square wave voltammogram was inversely related to logarithmic concentration of aflatoxin M1. Dynamic range of sensor was 1–10^5^ ppt aflatoxin M1. Sensor can be regenerated by treating electrode with 10% sodium dodecyl sulfate or 40 mM tris-HCl (pH 8.0) containing 10 mM ethylenediaminetetraacetic acid and 0.02% tween-20.

## Introduction

**Fig. 1:**
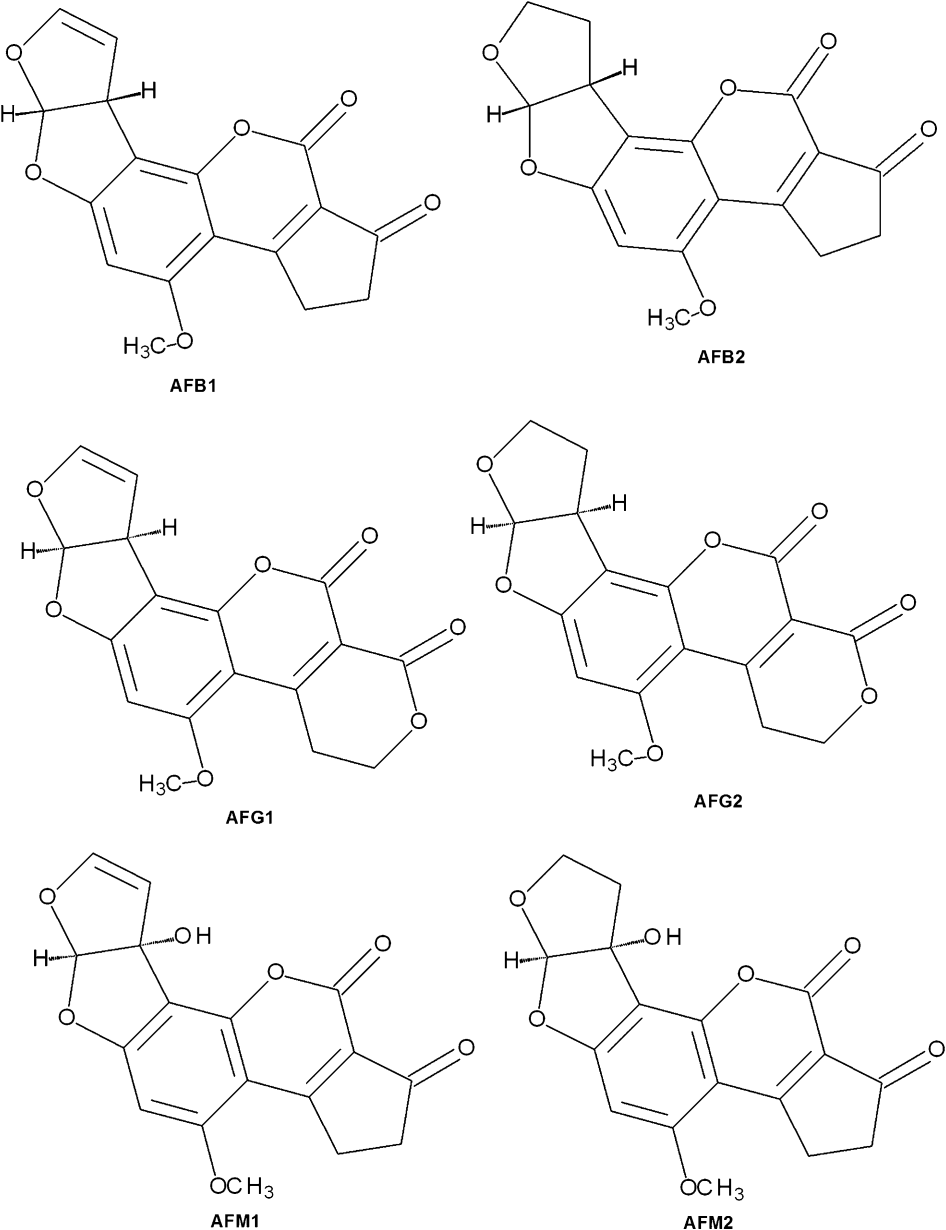
Structure of aflatoxins

Mycotoxins can result in carcinogenic, mutagenic and estrogenic effects and thus, these are hazardous for humans and animals. Mycotoxins can damage liver, kidney, lungs and cells involved in endocrine and immune functions (Bhatnagar et al. 2002). More than 300 mycotoxins including aflatoxins, ochratoxins, trichothecane, patulin are known (Sharma et al. 2017). These are metabolites produced by a number of fungi including *Acremonium*, *Alternaria*, *Aspergillus*, *Fusarium*, *Penicillium*, *Trichoderma* (Hussein and Brasel 2001) which can grow on grains (maize, wheat, barley), nuts (peanuts, groundnut) and fruits (apple, grape). Aflatoxins are most studied amongst all mycotoxins and these are produced by *Aspergillus parasiticus*, *Aspergillus flavus*, and rarely by *Aspergillus nomius* under hot and humid environment. Based on fluorescence properties, aflatoxins are grouped into B-group (blue fluorescence) or G-group (yellow-green fluorescence) while group M aflatoxins are metabolic products of group B aflatoxins and present in milk. G-group aflatoxins (G1and G2) have lactone ring while B (B1 and B2) and M-group (M1 and M2) aflatoxins have cyclopentene ring (Fig. 1). Amongst aflatoxin, aflatoxin B1 is the most common and highly toxic contaminant. Aflatoxin B1 (AFB1) and aflatoxin B2 are hydroxylated to form aflatoxin M1 (AFM1) and aflatoxin M2, respectively, in lactating animals. Biotransformation of AFB1 results in formation of AFM1, aflatoxin Q1, aflatoxin B1-exo-8,9-epoxide and aflatoxin B1-endo-8,9-epoxide. Aflatoxin B1-8,9-exo-epoxide is extremely electrophilic and covalently reacts with nucleophilic sites of either DNA or RNA or proteins. AFM1 and aflatoxins Q1 are less reactive with other molecules and are easily eliminated from the body in the urine (Wacoo et al. 2014).

AFM1 is classified as class 2B carcinogen. European Union has prescribed 50 ng AFM1/kg in liquid, dried or processed milk as maximum permissible level, while Codex Alimentarius Commission permits up to 500 ng AFM1/kg milk. Levels of mycotoxins can be measured by thin layer chromatography (Stubblefield and Shotwell 1981), high-performance liquid chromatography (Pathirana et al. 2010) and enzyme-linked immunosorbent assay (Rastogi et al. 2004). Although methods based on high performance liquid chromatography (HPLC) can provide confirmatory result, these methods require technical expertise and prior sample clean-up. Additionally, analysis of sample using these methods takes few to several hours. Enzyme-linked immunosorbent assay-based methods are relatively fast and are widely used for screening of samples. These methods require specific antibodies which are used as ligand for analytes. Aptamers are new class of ligand molecules which are widely used in developing methods for detection and estimation of analytes (Sun and Zu 2015).

Aptamers are single stranded DNA or RNA molecule generally comprising of less than 80 nucleotides and are selected from nucleic acid library ranging from 10^13^ to 10^15^ random sequences. These are selected through the process referred ‘Systematic Evolution of Ligand by Exponential Enrichment’ (Ellington and Szostak 1990; Tuerk and Gold 1990). These are new class of ligand molecules which can even surpass specificity of antibodies. These can even be generated against toxins (Huang et al. 2015; McKeague et al. 2014). These can be obtained in homogeneous form from commercial houses. Additionally, aptamer can be ligated with other molecules for obtaining biotinylated aptamer which makes use of streptavidin–biotin interaction in sensor design. Aptamers can be used to detect the presence of target molecules such as ochratoxin A (Bonel et al. 2011), tetracycline (Kim et al. 2010), tobramycin (Fernandez et al. 2011), diclofenac (Kheyrabadi and Mehrgardi 2012), lipopolysaccharide (Su et al. 2012), human immunodeficiency virus (Tombelli et al. 2005), β-casomorphin-7 (Parashar et al. 2015) etc. Aptamers can be immobilized on different surfaces for construction of electrochemical (Balamurugan et al. 2008; Kim et al. 2010; Sharma et al. 2017), quartz crystal microbalance (Le et al. 2013) and surface plasmon resonance (Vance and Sandros 2014) based sensing system.

Aptamers against AFM1 have also been generated (Dinckaya et al. 2011; Malhotra et al. 2014; Nguyen et al. 2013; Sharma et al. 2017; Pandey et al. 2017) and used for developing aptasensors. These sensors have low dynamic range and poor sensitivity. In present work, electrochemical sensor has been developed by immobilization of anti-aflatoxin M1 aptamer ‘AFAS3’ (Malhotra et al. 2014) conjugated with biotin and tetraethylene glycol (TEG) at its 3′-end on the gold electrode. Developed electrochemical aptasensor has dynamic range from 1 to 10^5^ ppt AFM1.

## Materials and methods

### Apparatus

Electrochemical analysis was performed at room temperature using electrochemical work station CHI 660 from CH Instruments, USA. The screen printed gold electrode (SPGE) comprising of gold working electrode (4 mm), gold counter electrode and silver reference electrode (DS220AT, DropSens, Spain) was integrated to work station.

### DNA aptamer and chemicals

**Fig. 2:**
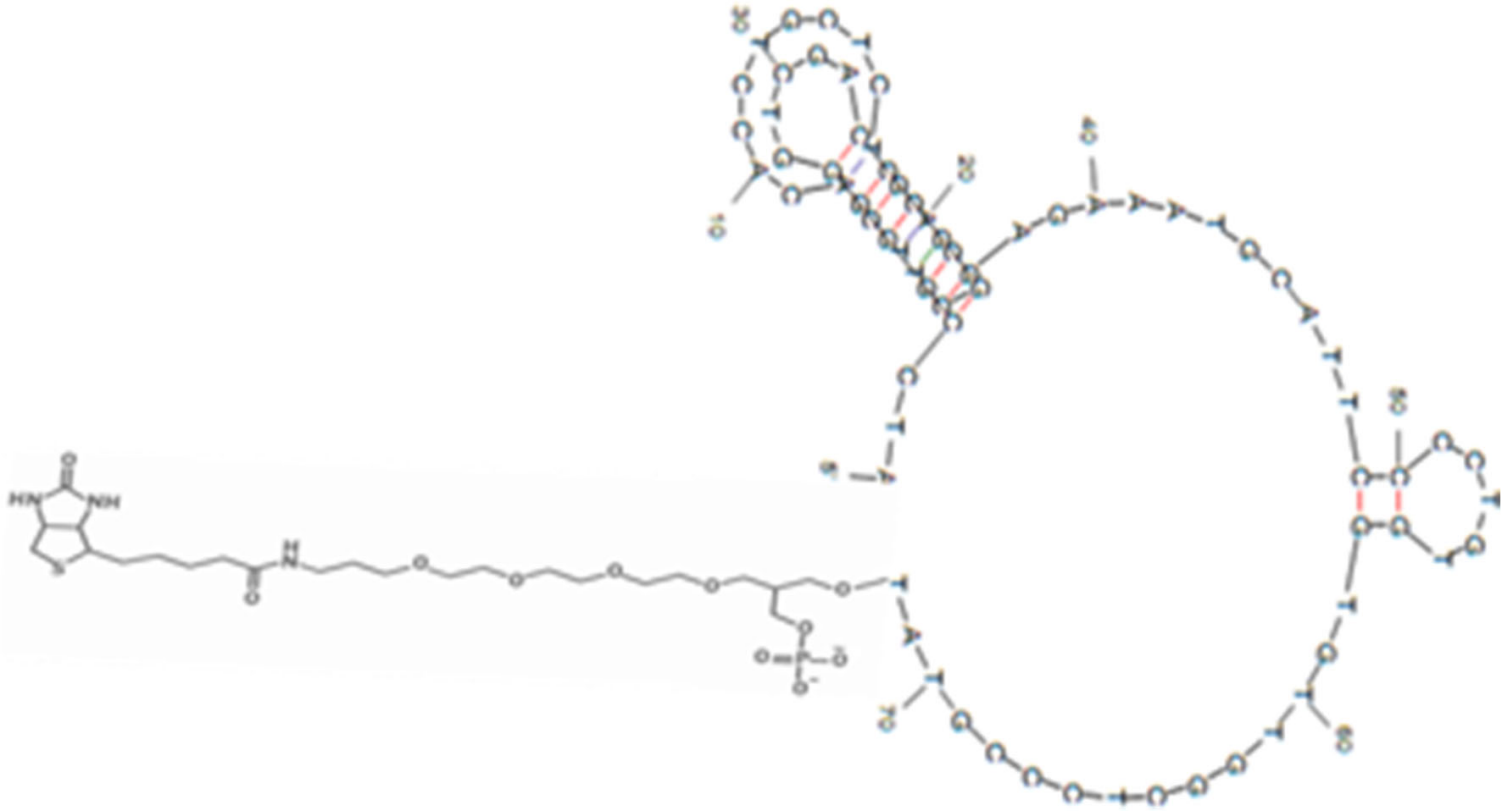
Secondary structure of the 3′ Biotin-tetraethylene glycol (TEG) modified aflatoxin M1 binding aptamer ‘AFAS3’

An AFM1 binding ssDNA aptamer ‘AFAS3’ (Malhotra et al. 2014) was extended by incorporating tetraethyleneglycol (TEG) and biotin at 3′-end of aptamer. The sequence of aptamer used was ^5^′ATCCGTCACACCTGCTCTGACGCTGGGGTCGACCCGGAGAAATGCATTCCCCTGTGGTGTTGGCTCCCGTAT-TEG-Biotin^3^′. The secondary structure of AFM-binding aptamer was predicted by Mfold programme (Fig. 2). HPLC purified aptamer was procured from Avantor Performance Materials India Ltd., Gurgaon, India. 3,3′-dithiodipropionic acid, and *N*-(3-dimethylaminopropyl)-*N*-ethylcarbodiimide (EDC) were from Sigma-Aldrich, USA. The *N*-hydroxysuccinimide (NHS) and streptavidin were purchased from Fluka, USA. Aflatoxins were procured from HiMedia Bioscience, India. Pure water having resistivity of 18.2 MΩ was used for electrode washing and sample preparation. All other chemicals used were of analytical grade.

### Immobilization of aptamer on gold electrode

**Fig. 3:**
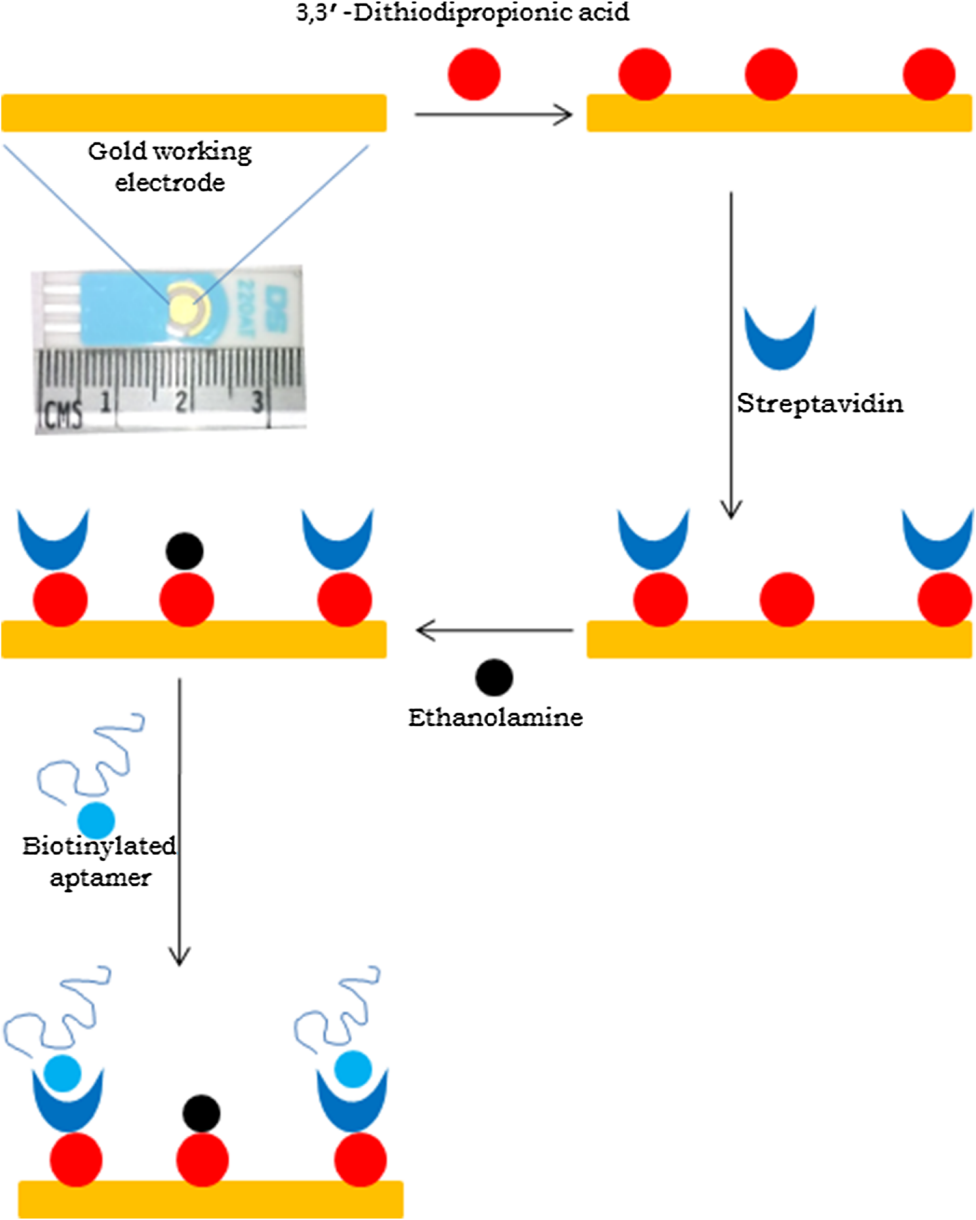
Schematic diagram of aptamer immobilization on screen printed gold electrode

Scheme for immobilization of aptamer on SPGE is depicted in Fig. 3. In the scheme, dithiodipropionic acid adsorbs to the gold surface forming self-assembled monolayer (Noll et al. 2006; Luczak 2009, 2011; Stobiecka et al. 2007). The carboxyl group of dithiodipropionic acid forms covalent bond with amino group of streptavidin protein or ethanolamine in presence of *N*-hydroxysuccinimide and 1-ethyl-3-(3-dimethylaminopropyl)-carbodiimide. Biotinylated aptamer binds streptavidin with non-covalent interactions.

SPGE was sequentially washed with 1 ml 10 mM of H_2_SO_4_ and 10 ml water. SGPE was then placed in humid chamber during all steps of immobilization except washing steps. 10 µl 200 mM 3,3′-dithiodipropionic acid (dissolved in ethanol) was layered over gold working electrode. After 30 min, all the three electrodes on SPGE were sequentially washed with 1 ml ethanol and 10 ml of water. The carboxylic groups of bound 3,3′-dithiodipropionic acid were activated by placing 10 µl 100 mM 4-morpholineethanesulfonic acid hydrate (MES) buffer (pH 6.0) containing 100 mM EDC and 1 mM NHS on the working electrode. After 1 h, electrodes were washed with 10 ml water. Then, 10 µl streptavidin (1 mg/ml) prepared in phosphate buffered saline (PBS), pH 7.5 was placed over working electrode and SPGE was incubated overnight at 4 °C. Then, electrodes were washed with 10 ml water. The free carboxyl groups of immobilized 3, 3′-dithiodipropionic acid on working electrode were blocked by incubating electrode with 10 µl 100 mM ethanolamine for 20 min. After draining of ethanolamine solution, 10 µl 2 nM biotinylated-TEG-aptamer (solubilised in water) was placed on working electrode. After 40 min, SGPE was washed with 10 ml water.

### Electrochemical analysis

Cyclic voltammetry (CV) was performed under the potential range of − 0.3–+ 0.8 V with a scan rate of 100 mV/s, and sample interval of 1 mV. Cyclic voltammograms were obtained at different stages of electrode modification viz., bare gold electrode, after dithiodipropionic acid coating, streptavidin coating and aptamer immobilization. Square wave voltammetry (SWV) was measured under potential range of + 0.8 to − 0.3 V with a frequency of 15 Hz, amplitude of 25 mV and incremental potential of 4 mV. All electrochemical analysis was performed at room temperature.

### Measurement of SWV under variable concentrations of aflatoxins and during regeneration

Aptamer-immobilized electrode was treated with known concentration (1, 10, 10^2^, 10^3^, 10^4^ and 10^5^ ppt) of AFM1 solubilised in tris buffer (20 mM tris-HCl, pH 7.6 containing 100 mM NaCl, 2 mM MgCl_2_, 5 mM KCl and 1 mM CaCl_2_) for 30 min at room temperature. After each treatment, the electrodes were washed with 3 ml tris buffer. Then, 100 µL 5 mM K_3_[Fe(CN)_6_] containing 0.1 M KCl was placed over all the three electrodes and square wave voltammetric response was measured. The electrode was sequentially incubated with AFM1 in its increasing order of concentration. Cross-reactivity of electrode was checked by comparing SWV response at 50 ppt aflatoxin B1 (AFB1) or 80 ppt AFB1 or 40 ppt AFM1 concentration. To remove bound AFM1 on electrode, the electrode was treated under either of the following conditions.

1. Two treatments of 30 s each with 6 M guanidine HCl or hot water (90 °C) or 10% sodium dodecyl sulfate (SDS).
2. One treatment of 15 min duration with warm buffer containing 40 mM tris-HCl (pH 8.0), 10 mM ethylenediaminetetraacetic acid (EDTA), 3.5 M urea and 0.02% tween-20.
3. One treatment of 15 min duration with 40 mM tris-HCl (pH 8.0), 10 mM EDTA and 0.02% tween-20.

SWV was recorded before and after treatment and also on electrode incubation withAFM1 solution.

## Results

### Immobilization of DNA aptamer on gold electrode

**Fig. 4:**
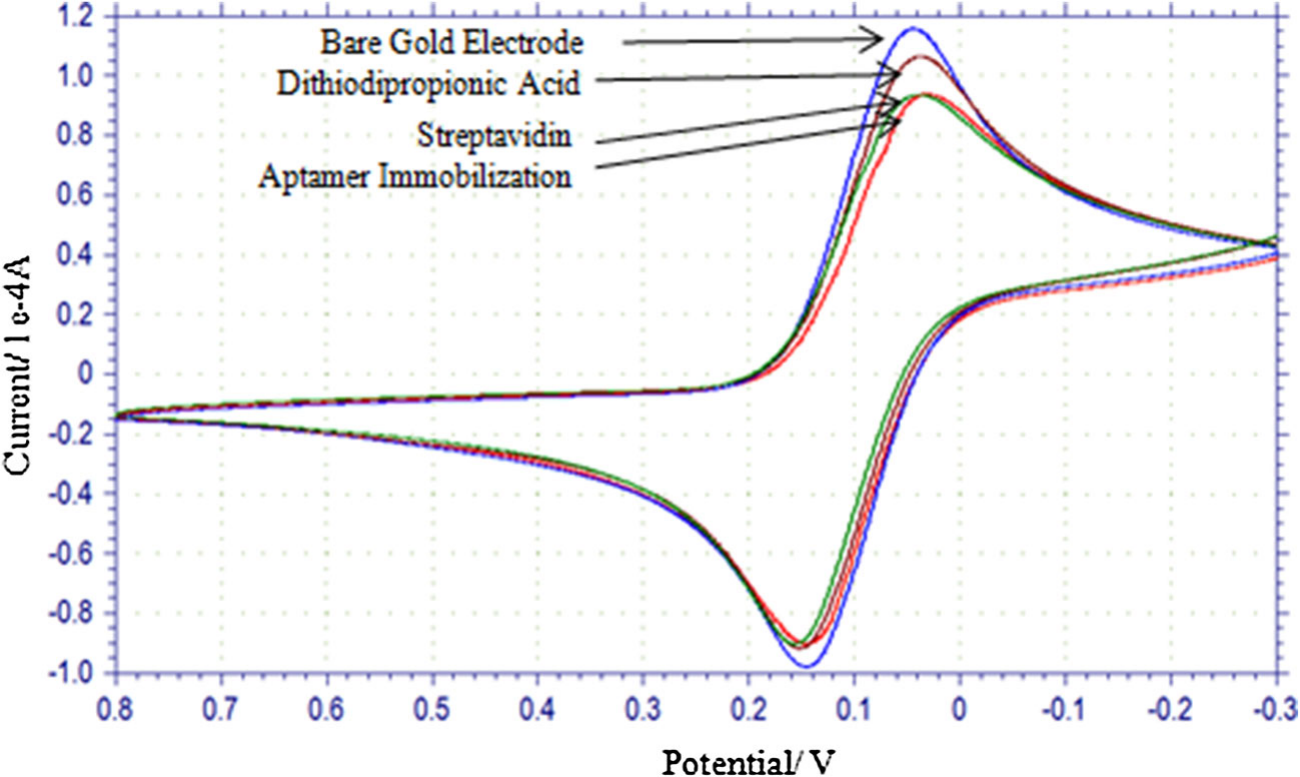
Cyclic voltammogram at different steps of immobilization of aptamer on gold working electrode

Cyclic voltammogram at different stages of immobilization process on electrode has been depicted in Fig. 4. When dithiodipropionic acid, streptavidin and aptamer were sequentially layered over the electrode surface, these resulted in lowering of peak current. This signifies that layers of dithiodipropionic acid, streptavidin and aptamer are formed on electrode.

### SWV Response at different concentrations of AFM1

**Fig. 5:**
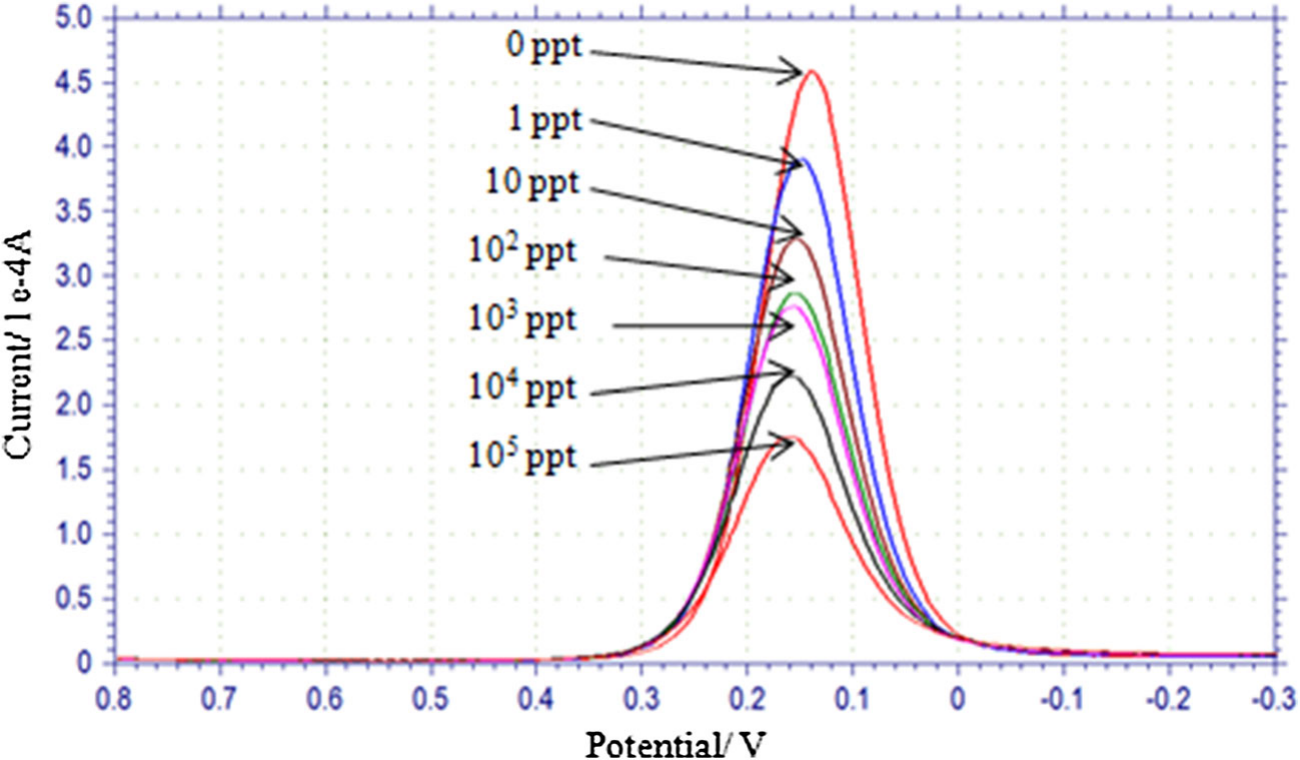
Square wave voltammogram of electrode at variable concentrations of aflatoxin M1 (AFM1). The concentration of AFM1 has been presented in parts per trillion (ppt)

**Fig. 6:**
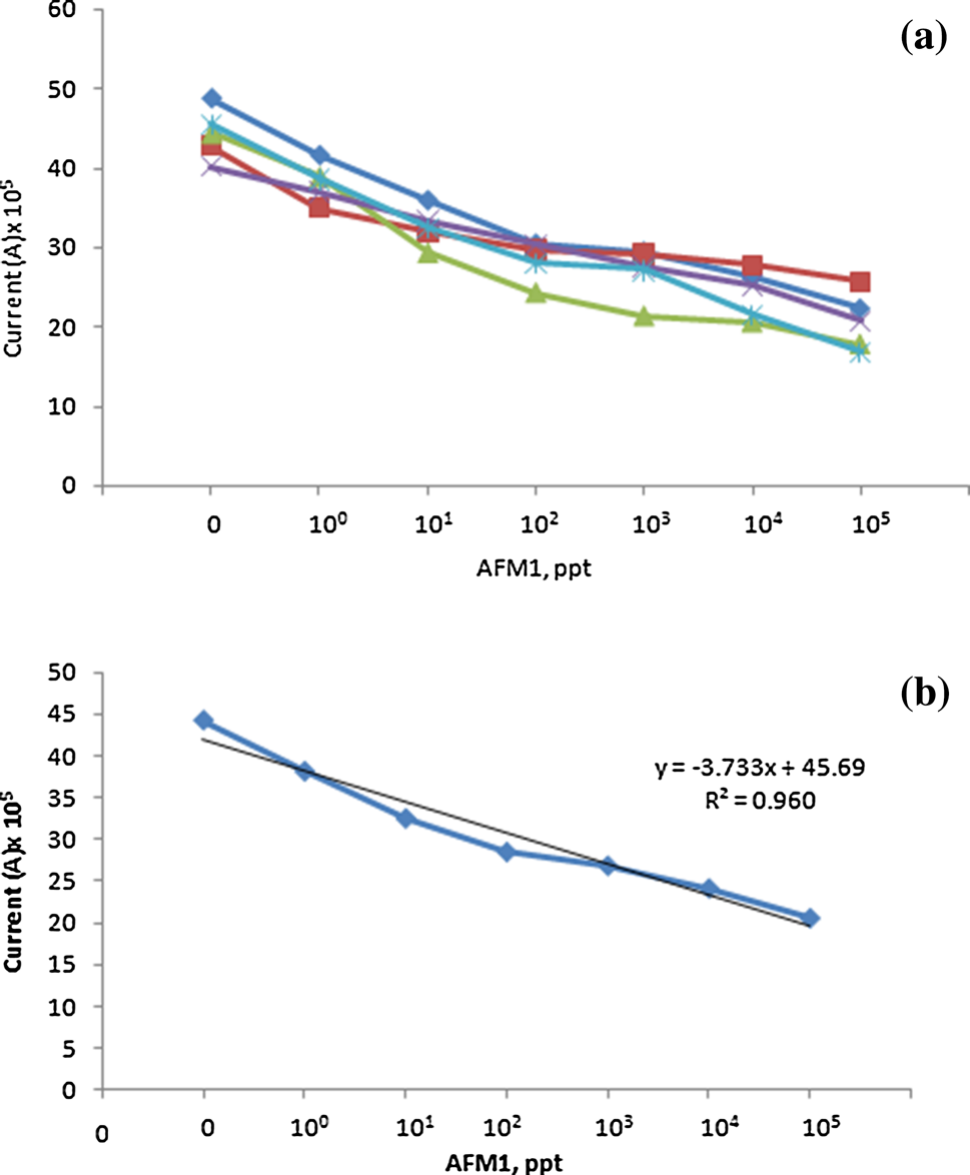
Peak current as a function of aflatoxin M1 (AFM1) concentration. **a** Each line represents response of individual electrode; **b** Mean values of peak current from five different electrodes. The concentration of AFM1 has been presented in parts per trillion (ppt)

SWV response of electrode on its incubation at 1, 10, 10^2^, 10^3^, 10^4^ and 10^5^ ppt concentration of AFM1 was measured (Fig. 5). As the concentration of AFM1 was increased, peak current decreased. Aptamer immobilization was performed on five different SGPE and response of modified electrodes at different concentrations of AFM1 was similar. The data on peak current *vis*-*à*-*vis* different concentrations of AFM1 for five different electrodes is shown in Table 1 and plotted in Fig. 6a. There was progressive decrease in current with increase in AFM1 concentration. When mean values of current were plotted against logarithmic concentrations of AFM1, a linear relationship with *R* ^2^ = 0.960 was obtained (Fig. 6b). There was variation up to 20% in peak current measured on five different electrodes in absence of AFM1 (Table 1).

**Table 1:** Peak current (A) × 10^5^ of five modified electrodes on their incubation with variable concentration of AFM1

AFM1 Conc., ppt	Current (A) × 10^5^					
	Electrode					Mean ± SD
	1	2	3	4	5	
0	48.75	42.73	44.43	40.04	45.44	44.28 ± 3.22
1	41.59	34.98	38.89	36.89	38.57	38.18 ± 2.45
10	35.91	32.01	29.29	33.23	32.36	32.56 ± 2.38
10^2^	30.44	29.7	24.24	30.27	28.08	28.55 ± 2.58
10^3^	29.44	29.14	21.3	27.55	27.1	26.90 ± 3.28
10^4^	26.06	27.7	20.52	25.2	21.54	24.20 ± 3.05
10^5^	22.24	25.69	17.73	20.71	16.82	20.64 ± 3.57

### Specificity of EC aptasensor for AFM1

**Fig. 7:**
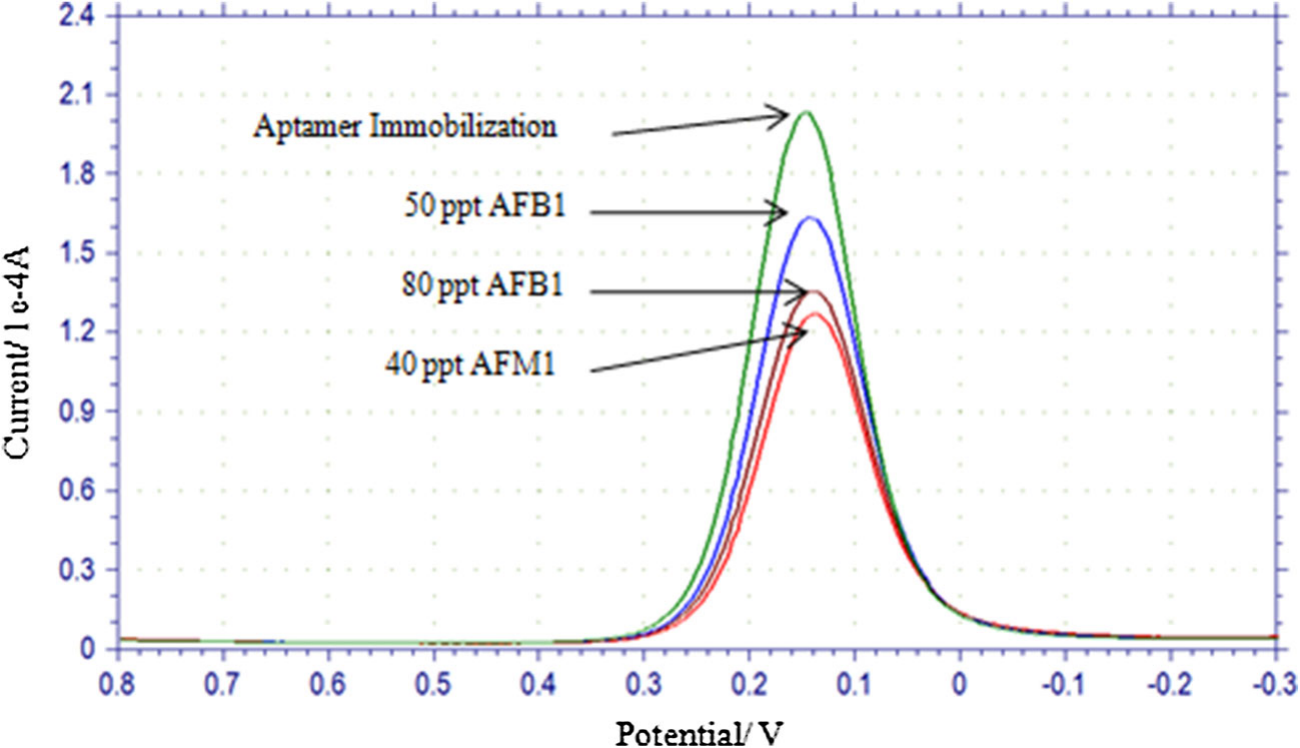
Cross reaction of aptamer ‘AFAS3’ with aflatoxin B1 (AFB1). Electrode response was checked with 50 and 80 parts per trillion (ppt) AFB1 and 40 ppt aflatoxin M1 (AFM1)

Cross-reactivity of aptamer ‘AFAS3’ with AFB1 was also checked by measuring SWV at 50 ppt AFB1, 80 ppt AFB1 or 40 ppt AFM1 (Fig. 7). At both the concentrations (50 and 80 ppt) of AFB1, current dropped in measurable amounts. This shows that AFB1 is bound to aptamer on electrode. However, drop in current was higher with 40 ppt AFM1 in comparison to drop observed with either 50 or 80 ppt AFB1 concentration. This proves that aptamer has comparatively more affinity towards AFM1.

### Regeneration of the modified electrode surface

**Fig. 8:**
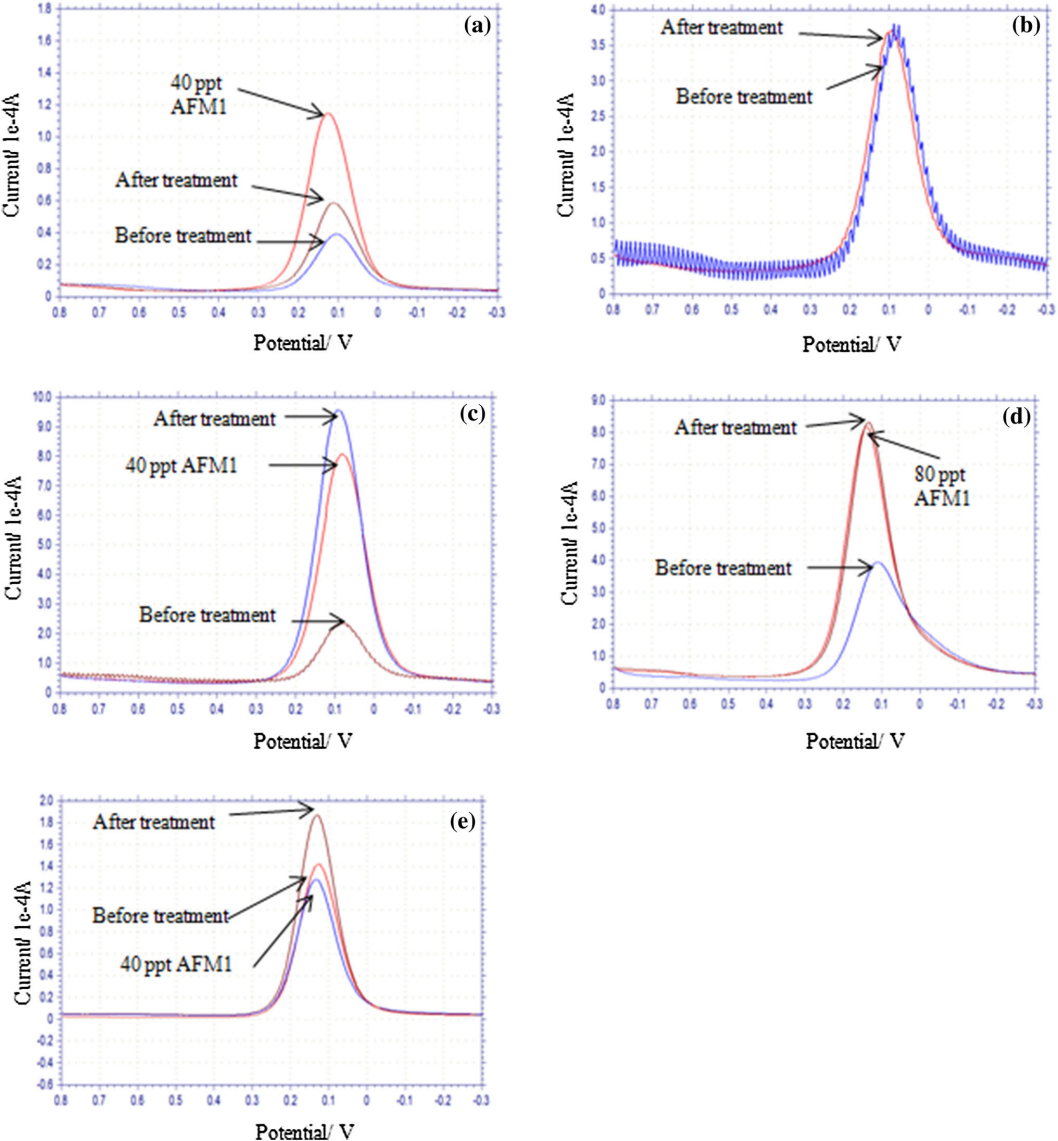
Regeneration of electrode with **a** 6 M Guanidine-HCl;** b** hot water (90 °C); **c** 10% sodium dodecylsulphate;** d** 40 mM tris-HCl (pH 8.0) containing 10 methylendiamine tetra acetic acid, 3.5 M urea and 0.02% tween-20 and** e** 40 mM tris-HCl containing 10 mM EDTA and 0.02% tween-20. For details, see material and method section. Electrode response was checked before treatment of electrode with regeneration solution, after regeneration treatment and on exposure to indicated concentration of aflatoxin M1 (AFM1) in parts per trillion (ppt)

Aptamer binds with target molecule with high affinity and therefore, removal of bound target molecule is difficult under ordinary washing protocols. Further, washing protocols should ensure complete removal of target leading to restoration of peak current. Regeneration of electrode was attempted with 6 M guanidine HCl (Fig. 8a), hot water (Fig. 8b), 10% SDS solution (Fig. 8c), 40 mM tris-HCl (pH 8.0) containing 10 mM EDTA, 3.5 M urea, 0.02% tween-20 (Fig. 8d) and 40 mM tris-HCl (pH 8.0) containing 10 mM EDTA and 0.02% tween-20 (Fig. 8e) under the defined conditions (see materials and methods). Incubation of electrode with 10% SDS solution resulted in substantial increase in peak current indicating removal of bound AFM1. Peak current decreased on incubation with 40 ppt AFM1 (Fig. 8c). Thus, the electrode can be regenerated with 10% SDS solution. Although peak current increased after incubation of electrode with 40 mM tris-HCl (pH 8.0) containing 10 mM EDTA, 3.5 M urea, 0.02% tween-20 (Fig. 8d), there was no change in electrode response on incubation with AFM1. This indicated that regenerated electrode lost the capacity to bind with AFM1. Regeneration with 40 mM tris-HCl, pH 8.0 containing 10 mM EDTA, 0.02% tween-20 also resulted in increase in peak current (Fig. 8e) which is a sign of removal of target molecules. On incubation with 40 ppt AFM1, peak current decreased (Fig. 8e) and this indicates binding of AFM1 to regenerated electrode. Regeneration with 6 M guanidine HCl (Fig. 8a), hot water (Fig. 8b) and tris-HCl containing urea (Fig. 8d) did not work as peak current did not increase on treatment with hot water (Fig. 8b) or peak current did not drop on incubation of electrode with AFM1 solution (Fig. 8a, d).

**Fig. 9:**
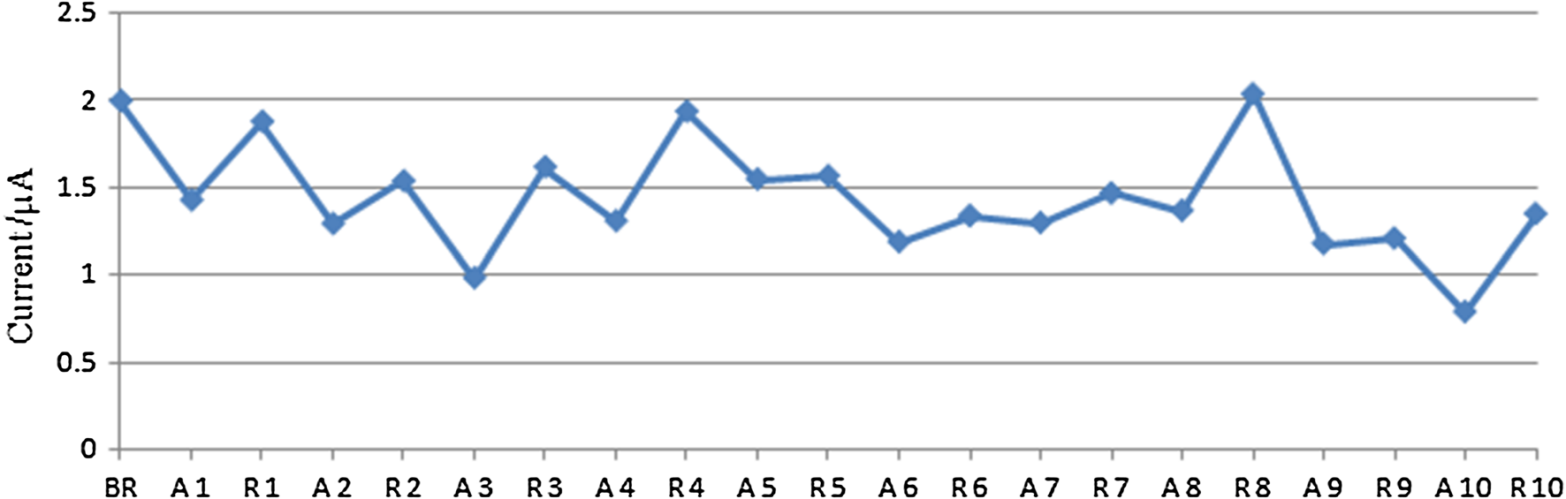
Regeneration of electrode. Electrode was regenerated 10 times. After each treatment of electrode with regeneration buffer (40 mM tris-HCl containing 10 mM EDTA and 0.02% tween-20) and 40 part per trillion (ppt) aflatoxin M1 (AFM1), peak current was recorded. BR, before regeneration; A, treatment with AFM1; R, treatment with regeneration buffer. Numerical values suffixed to A and R on *x*-axis indicate number of regeneration cycles

After attempting different protocols for regeneration of electrode, efforts were made to see possibility of repeated regenerations on incubation of electrode with 40 mM tris-HCl, pH 8.0 containing 10 mM EDTA, 0.02% tween-20. After each regeneration, electrode was treated with 40 ppt AFM1. Peak current after each regeneration treatment and after each exposure to 40 ppt AFM1 are shown in Fig. 8. In total, 10 such cycles (regeneration and treatment with 40 ppt AFM1) were attempted. Treatment of electrode with regeneration buffer in cycle 1, 2, 3, 4, 8 and 10 resulted in increase in peak current. On two occasions (cycle 4 and 8), peak current enhanced to original value (Fig. 9). There was decrease in peak current after first, second, third, fourth, fifth, eighth and ninth regeneration cycle after AFM1 treatment. This indicates that regenerated electrode has interacted with AFM1. The protocol using 40 mM tris-HCl pH 8.0 containing 10 mM EDTA, 0.02% tween-20 appears to be promising for regeneration of the electrode for sensing of AFM1.

## Discussion

An electrochemical system provides interplay between chemical reaction and electricity. It measures chemical reaction in terms of electrical current or potential. The system uses three electrodes comprising of working electrode, reference electrode and counter electrode. When constant potential is applied between working and reference electrodes, oxidation–reduction reaction gives an increase in current which is proportional to concentration. The current is measured between working and counter electrode (Wang 2006). The current signal at the surface of electrode is produced by transfer of electrons from electrode to redox species. Modified electrode used in present study results in increased mass transfer on electrode after binding to AFM1 and this results in increased resistance in movement of redox probe [potassium ferricyanide K_3_Fe(CN)_6_] and ultimately its oxidation reduction reaction. In this situation, current will decrease on increase in concentration of analyte. In our studies using modified electrode, peak current decreased with increase in concentration of AFM1. Relationship between peak current and dose (AFM1 concentration) was established. Diminished current with increased AFM1 concentration suggests that AFM1 created impedance and electrochemical signal has arisen from mass transfer effects. The electrochemical signal was linear and inversely related to logarithmic concentration of AFM1.

Present scheme of immobilization uses two different strong interaction. Binding of sulphur to gold is strong (Xue et al. 2014) and thus dithiodipropionic acid will be not washed off during repeated use of immobilized electrode. Streptavidin and biotin interaction surpass affinity of antigen–antibody interaction and therefore, biotinylated aptamer will also not be washed off during repeated use. Possible drawback in this scheme of immobilization could be from interference from biotin and sulphur containing molecules. Possibility do exist that biotin in biological sample at reasonable concentration can partly remove immobilized biotinylated aptamer.

Recent trend indicates that electrochemical sensor based assay protocols for aflatoxins including AFM1 are actively pursued (Sharma et al. 2017). This includes differential pulse voltammetry (Ammida et al. 2004), chronoamperometry (Micheli et al. 2005), intermittent pulse voltammetry (Piermarini et al. 2007; Bacher et al. 2012), electrochemical impedance spectroscopy (Vig et al. 2009) and linear sweep voltammetry (Tan et al. 2009). Present method provides dynamic range from 1 ppt to 10^5^ ppt for AFM1. Sensitivity of the present method and that of label free impedimetric immunosensor (Bacher et al. 2012) is equal to 1 ppt which is better than 25 ppt observed with chronoamperometric sensor (Micheli et al. 2005) or 30 ppt reported with differential pulse voltammetry (Ammida et al. 2004) and intermittent pulse voltammetry (Piermarini et al. 2007). A non-enzymatic nanomagnetic electro-immunosensor capable of detecting 0.2 ppt aflatoxin B1 has been fabricated and the sensor requires antibody (Masoomi et al. 2013). This sensor can be regenerated by an external magnetic field and design can be applied for any analyte. However, fabrication involves multiple steps and requires antibodies.

Use of aptamers in developing methods for AFM1 assay is leading to desired sensitivity and improved dynamic range (Guo et al. 2016). Our results are comparable with Guo et al. (2016) and are better than other AFM1 aptasensors (Dinckaya et al. 2011; Nguyen et al. 2013). The method described by Guo et al. (2016) uses ‘AFAS3’ aptamer in development of quantitative PCR for AFM1. The aptamer ‘AFAS3’ was generated by Malhotra et al. (2014) and have been used in present work as well as by Guo et al. (2016). Aptamer ‘AFAS3’ does not exhibit cross reaction with ochratoxin A, zearalenone and fumonisin, but results in negligible interference from aflatoxin B1 and aflatoxin B2 (Malhotra et al. 2014; Guo et al. 2016).

The aptamer used in present work has spacer ‘TEG’ and linker ‘biotin’. The binding of biotin with streptavidin, although non-covalent, is very strong (*K*_d_ value in the range of 10^−15^ nM) and this will enable attachment of aptamer to streptavidin during repeated use of sensor. TEG provides 15 atom spacer arm thereby making binding sites of aptamer freely available for interaction with AFM1. In our laboratory, AFAS3 tagged with only biotin failed to recognize aflatoxin M1-peroxidase in enzyme-linked aptamer sorbent assay. This further strengthens the view that spacer like TEG will provide flexibility to biotinylated aptamers for its binding with target.

Guanidine-HCl and urea can denature proteins. In present work, it appears that during regeneration of electrode with guanidine-HCl and urea, the conformation of biotin binding pockets in streptavidin might be altered and biotinylated aptamer is detached. Regeneration of electrode with 10% SDS or 40 mM tris-HCl (pH 8.0) containing 10 mM EDTA, 0.02% tween-20 can be preferred whenever biotin–streptavidin interaction is exploited for immobilization of aptamer on electrode.

## Conclusion

An aptamer-based electrochemical biosensor was designed and validated. The sensor can detect trace amount of AFM1 in solution and has large dynamic range from 1 to 10^5^ ppt. Immobilization of aptamer involving avidin–biotin and sulphur–gold interactions and use of spacer ‘TEG’ in aptamer appears to improve stability of sensor and flexibility of aptamer in recognizing AFM1.
